# Impact of extracellular alkalinization on the survival of human CD24^-^/CD44^+^ breast cancer stem cells associated with cellular metabolic shifts

**DOI:** 10.1590/1414-431X20176538

**Published:** 2017-07-17

**Authors:** S.I. Wanandi, I. Yustisia, G.M.G. Neolaka, S.W.A. Jusman

**Affiliations:** 1 Department of Biochemistry and Molecular Biology, Faculty of Medicine, Universitas Indonesia, Jakarta, Indonesia; 2 Department of Biochemistry, Faculty of Medicine, Hasanuddin University, Makassar, Indonesia

**Keywords:** Extracellular alkalinization, Metabolic states, Breast cancer stem cells, Cell survival, NaHCO_3_

## Abstract

Cancer stem cells reside in a distinct region within the tumor microenvironment that it is believed to play a fundamental role in regulating stemness, proliferation, survival, and metabolism of cancer cells. This study aimed to analyze the effect of extracellular alkalinization on metabolism and survival of human CD24^-^/CD44^+^ breast cancer stem cells (BCSCs). BCSCs were cultured in alkalinized DMEM-F12 and incubated at 37°C, 5% CO_2_, and 20% O_2_ for 30 min, 6, 24, and 48 h. After each incubation period, we analyzed the modulation of various mRNA expressions related to pH and cellular metabolic regulation using the qRT-PCR. Metabolic state was measured using colorimetric and fluorometric assays. To examine cell proliferation and apoptosis, we used trypan blue and annexin V/propidium iodide assay, respectively. This study demonstrated that alkalinization could stimulate extracellular carbonic anhydrase (CAe) activity, as well as CA9 and HIF1α expression. Under alkaline pH and HIF1α regulation, glucose consumption, extracellular lactate production, and LDH activity of BCSCs were upregulated while O_2_ consumption was downregulated. These metabolic shifts seemed to promote apoptosis and suppress the proliferation of BCSCs. To conclude, modulation of the extracellular environment through alkalinization could change the metabolic states of BCSCs, which in turn affect the cell survival.

## Introduction

The tumor microenvironment is the environment surrounding the cancer cells, which consists of stromal cells, non-cellular components and various kinds of soluble factors forming the extracellular matrix. The tumor microenvironment also has distinctive physical and chemical parameters such as oxygen pressure (O_2_), pH, interstitial pressure and fluid flux (1–3). The microenvironment has a major role in regulating stemness, proliferation, tenacity against apoptosis, and it also has a protective function that keeps the cancer stem cells (CSCs) safe from genotoxic damage (4,5).

It is known that the microenvironment of solid tumors tends to be hypoxic, which may affect their metabolism (4). *In vitro* studies conducted by Vlashi et al. (6,7) have demonstrated that the CSCs of glioma and breast cancer are dependent on oxidative phosphorylation for energy metabolism; while its differentiated progeny has shown a more glycolytic phenotype. Feng et al. (8) reported that breast cancer stem cells (BCSCs), also called tumor initiating cells, preferentially performed a glycolytic phenotype over oxidative phosphorylation compared to non-tumorigenic cells. The contradiction of these results indicates that further studies on the metabolic states of BCSCs are essential considering the crucial role of the cell population in carcinogenesis and the central role of metabolism on CSCs survival.

One of the metabolic consequences of hypoxia is tumor acidity. Unlike healthy tissues, cancer has higher intracellular pH (pHi, ≥7.4) and lower extracellular pH (pHe) with a range of 6.7–7.1 (9,10). The pH of tumor tissue has an important role in the survival of cancer cells and their malignant characteristics. The slightly basic pH (≥7.4) has several roles in increasing cell proliferation, preventing apoptosis and cytoskeletal changes in cell migration. It has been reported that the increase of extracellular acidity could facilitate cell invasion, modulate cell binding to the extracellular matrix, and increase cellular protease activity, hence, suggesting its essential role in cancer metastasis (9,11). The specific characteristic of cancer acidity and its various consequences on survival and spreading of cancer cells has become a prospective and strategic approach to a novel cancer treatment.

Much effort has been carried out to increase the effectiveness of cancer therapy including the development of therapeutic targets on CSCs. However, the stemness, tumorigenicity, dormancy, and plasticity of CSCs are obtained from the interaction of these cells with their environment. Therefore, the development of a targeted therapy must also consider the CSC microenvironment factors. To study the effect of microenvironment pH changes on the survival of BCSCs, we conducted a study that modulated the pHe of human BCSCs using the alkalinizing agent sodium bicarbonate (NaHCO_3_). We hypothesized that alkaline pHe might alter the metabolic preference, leading to the decrease of CSCs survival.

## Material and Methods

### Culture of human breast cancer stem cells (BCSCs)

In our previous study, primary culture of human breast cancer were sorted using magnetic-activated cell sorting conjugated with anti-CD24 and anti-CD44 antibody resulting in CD24^-^/CD44^+^ cells for BCSCs and CD24^-^/CD44^-^ cells for non-BCSCs (Patent registration from the General Directorate of Intellectual Property Right, Ministry of Law and Human Right, Republic of Indonesia; No. P0021300369).

BCSCs were cultured in serum free DMEM/F12 medium in 15 mM HEPES buffer supplemented with 1% penicillin/streptomycin, 1% amphotericin B (250 µg/L, 0.2% gentamycin sulfate (50 mg/mL), and 14.5 mM NaHCO_3_ under standard condition (at 37°C in a humidified atmosphere of 5% CO_2_ and 20% O_2_). Although there are no specific growth factors for stem cells, the human BCSCs (CD24^-^/CD44^+^ cells) still retain their pluripotency and tumorigenicity under this culture condition, as indicated by higher Oct-4 and ALDH1 mRNA expression, as well as higher mammosphere forming unit compared to their counterpart CD24^-^/CD44^-^cells and the unsorted primary breast cancer cells (Patent registration from the General Directorate of Intellectual Property Right, Ministry of Law and Human Right, Republic of Indonesia; No. P00201607099).

### Alkalinization of culture medium using NaHCO_3_

BCSCs were initially seeded at 5×10^5^ cells/well in 6-well plates and cultured in 3 mL/well of DMEM-F12 standard medium, pH 7.4, at 37°C, 5% CO_2_ and 20% O_2_ for 24 h. Afterwards, the extracellular alkalinization was performed by replacing the initial BCSCs medium with standard medium supplemented with various volumes of 8.4% NaHCO_3_ (Meylon-84®, Otsuka, Indonesia) to generate the final concentration of 10, 30, 50, 75, and 100 mM. The cells were then incubated in the alkalinized medium at 37°C, 5% CO_2_ and 20% O_2_ for 0, 0.5-, 6-, 24-, and 48-h. After each incubation period, pH of cell culture medium (pHe) was immediately measured. To remove dead cells, cultured cells were then harvested by centrifugation at 200 *g* for 10 min at room temperature. Cell pellet and culture supernatant were collected for various analysis. Cell pellet containing viable cells were re-suspended in PBS buffer. Viable cell number and percentage of viable cells to total cells (cell viability) were determined using trypan blue exclusion assay in automated cell counter (Luna®, Logos Biosystems Inc., Korea). The remaining cells collected in pellets after NaHCO_3_ treatment still have high percentage of viability (>90%) similar to control without treatment, indicating that the experimental data were obtained from the remaining viable cells.

### pHe measurement

pHe was determined by measuring pH of the culture medium using pH electrode with Micro Bulb for 96-well plate (Hanna®) connected with pH meter (HI 2210®, Hanna). After incubation, 300 µL medium from each alkalinized and non-alkalinized BCSC culture was collected in a test tube for pH measurement. The pH should be immediately measured upon removing the cell culture plate from the incubator, since changes of CO_2_ concentration and temperature could affect the results.

### Quantitative reverse transcription-PCR

Total RNA was extracted from cell pellets using Tripure® RNA Isolation Kit (Roche, Germany) according to the manufacturer's protocol. Total RNA concentration was quantified using spectrophotometer (Varioskan Flash®, Thermo Scientific, Finland). Samples with an A260/A280 ratio of 1.6–2.0 were considered to be free of DNA and proteins. Quantitative RT-PCR was performed using KAPA SYBR Fast® qPCR (Kapa Biosystems, USA) in the Exicycler^™^ 96 (Bioneer, Korea). The PCR primers for CA9, HIF1α, GLUT1, and 18s RNA are listed in Table 1. All primers have been tested for their efficiency, resulting in a high primer efficiency for all genes (>95%). Ct for each gene was determined, and ΔΔCt was normalized to the designated reference sample. Gene expression values were then relatively calculated using the Livak method (2^−ΔΔCt^).

**Table 1.** Primers used in qRT PCR.

Genes	Primers	Amplicon (bp)
CA9	Sense 5′-GGCTACAGCTGAACTTCCGA-3′ Antisense 5′-GCCAAAAACCAGGGCTAGGA-3′	155
GLUT1	Sense 5′-GCTTCCAGTATGTGGAGCAAC-3′ Antisense 5′-GGTCCGGCCTTTAGTCTCAG-3′	116
HIF1	Sense 5′- GGCGCGAACGACAAGAAAAAG -3′ Antisense 5′- AGTGGCAACTGATGAGCAAG-3′	122
18S RNA	Sense 5′- AAACGGCTACCACATCCAAG-3′ Antisense 5′-CCTCCAATGGATCCTCGTTA-3′	155

### Extracellular carbonic anhydrase (CAe) activity

Cultured BCSCs of 10^6^ cells were centrifuged at 200 *g* for 10 min at room temperature. The obtained pellet containing intact cells was resuspended with 20 mM Tris-sulfate buffer, pH 8.3, and the extracellular carbonic anhydrase enzyme activity were immediately measured using the titrimetric method of Wilbur and Anderson with modification according to Rigobello-Masini et al. (12), i.e., 1.5 mL of saturated CO_2_ deionized water was added to 3 mL of BCSCs resuspended in Tris-sulfate buffer or buffer without cells. The reaction was performed on ice at a temperature maintained between 0 and 4°C. The time required (in s) for the saturated CO_2_ deionized water to lower the pH of Tris-sulfate buffer without cells (as the blank; T0), and Tris-sulfate buffer containing intact BCSCs (T) from 8.3 to 6.3 was measured, and the enzymatic activity was calculated using the following equation: A unit of activity = (T_0_/T) – 1

### Glucose consumption, lactate production, and LDH activity assay

Glucose consumption was determined by subtracting the glucose concentration in DMEM-F12 (17.5 mM) before incubation with that remained in the medium after the incubation period. The glucose level was measured using O-toluidine colorimetric assay (Sigma-Aldrich, USA) and spectrophotometer at 625 nm.

To determine the extracellular lactate level, the amount of lactate present in the supernatant of BCSC culture was determined using the L-Lactate Assay Kit (Abcam, UK) and spectrophotometer at 450 nm. The intracellular lactate level was measured in the lysate of cell pellets using Lactate Colorimetric/Fluorometric Assay Kit (BioVision, USA).

Lactate dehydrogenase (LDH) activity was analyzed using a Lactate Dehydrogenase Activity Assay Kit (Sigma) in cell lysate, which represents the intracellular LDH. We also measured the extracellular LDH activity in the cell culture supernatant to calculate the LDH release for the determination of cell viability (see below).

### Extracellular oxygen concentration assay

Oxygen consumption was determined using sensitive phosphorescent probes that were quenched at excited state in the presence of oxygen *(*Extracellular O_2_ probe, Abcam*)*. Briefly, after culturing BCSCs for an incubation period, cells were harvested and then transferred to a fluorescence 96-well plate with a density ∼8×10^4^ cells/well. The 10-µL probes from 1 μM stock solution were dispensed into each well, and 100 μL of preheated mineral oil (30°C) was added to each well to enhance the assay sensitivity by minimizing interference from ambient oxygen. Probe signals were measured in a fluorescence plate reader equipped with a time-resolved mode preset to 37°C at 1.5 min intervals for 60 min using excitation and emission wavelengths of 380 nm and 650 nm, respectively.

### HIF1α protein level

Total protein was extracted from 5×10^5^ cells. HIF1α protein level was assayed using HIF1A Human ELISA kit (Abcam) according to the manufacturer's protocol. Data are reported per total protein.

### Inhibition of HIF1α expression using FM19G11

FM19G11 inhibits HIF1α expression, thus represses the transcriptional activity of its target genes (13). Cells were simultaneously treated with FM19G11 and 100 mM NaHCO_3_ and incubated for 24 h. After treatment, pHe and cell viability (ratio of viable cell to total cell number) was determined using trypan blue exclusion assay on the automated cell counter. The expression of HIF1α and GLUT1 mRNA were analyzed using qRT-PCR method, as described above. Protein expression of HIF1α was analyzed using Human/Mouse HIF-1α Total Immunoassay kit (R&D System, USA) according to the manufacturer's protocol.

### Cell viability and population doubling time (PDT) assay

To determine cell viability, we applied trypan blue exclusion assay. Viable cells were counted using an automated cell counter (Luna®, Logos Biosystems Inc.). Cell viability was also examined using the calculation of percent LDH release obtained from the ratio between LDH activity in supernatant (extracellular) and in cell lysate (intracellular). Extracellular LDH activity represents the presence of this enzyme released by lysed cells, hence this method could be used to determine the cytotoxicity as previously described by Gloria et al. (14).

Population doubling time defines the average time taken by a cell population to double in the log-phase/exponential phase. To determine the PDT of BCSCs, about 5×10^5^ cells were grown under standard conditions with or without alkalinization for 48 h. Cells were counted every 6 h using trypan blue exclusion assay. According to Davis (15), the formula used was (t_2_ - t_1_)/3.32 (log n_2_ - log n_I_), where t is time and n number of cells.

### Apoptosis assay

Apoptosis test was performed using Annexin V-FITC Apoptosis Detection Kit (Abcam). About 5×10^5^ BCSCs were harvested and rinsed with cold PBS twice. The next steps were carried out following the protocol instructions.

### Statistical analysis

All data are reported as means±SE of at least triplicates. A P-value of <0.05 in the independent *t*-test and one-way ANOVA was considered to be statistically significant.

## Results

### BCSC response toward increased pHe

**Figure 1.**
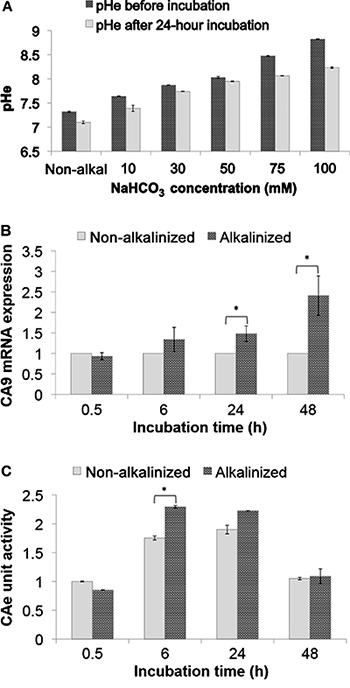
*A*, pHe of the breast cancer stem cells culture medium measured by a micro bulb pH electrode at various concentrations of NaHCO_3_ before and after 24-h incubation at 37°C with 5% CO_2_. *B*, qRT-PCR analysis of CA9 mRNA expression after alkalinization with 100 mM NaHCO_3_. *C*, CAe activity measurement using the Wilbur Anderson method. Data are reported as means±SE for n=9. *P<0.05 non-alkalinized *vs* alkalinized BCSCs CD24^-^/CD44^+^ culture (*t*-test). CAe: carbonic anhydrase; pHe: extracellular pH.

Following the alkalinization of BCSC cultures using various NaHCO_3_ concentrations, we showed that the increased pHe was consistent with the increase of NaHCO_3_ concentration (Figure 1A). The addition of 100 mM NaHCO_3_ resulted in the highest increase of pHe (8.84±0.00); however, the pHe was gradually decreased along with the increase of NaHCO_3_ incubation period. This result was consistent with the pH of control medium without cells, which was also gradually reduced after certain periods of NaHCO_3_ incubation. We assume that the CO_2_ gas in the incubator became soluble in the culture medium and produced carbonic acid, which could lower the pH of the medium. Therefore, to analyze the pattern of reduced pHe, the pH of control medium (without cells) was subtracted by the pHe of BCSC culture known as the ΔpH (Table 2). This study demonstrated that ΔpHe of BCSCs after 6- to 48-h alkalinization was significantly higher compared to that of the control without alkalinization (P≤0.05).

**Table 2.** Comparison of ΔpHe of breast cancer stem cells with and without alkalinization with NaHCO_3_.

Incubation time (h)	Without alkalinization			Alkalinization with NaHCO3			P value
	pH of control medium	pHe of BCSC CD24^-^/CD44^+^ culture	ΔpHe	pH of control medium	pHe of BCSC CD24^-^/CD44^+^ culture	ΔpHe	
0	7.40±0.01	7.45±0.01	-0.05±0.00	8.86±0.03	8.84±0.00	0.02±0.03	0.25
0.5	7.27±0.01	7.38±0.01	-0.11±0.01	8.46±0.01	8.55±0.01	-0.09±0.00	0.43
6	7.31±0.01	7.09±0.00	0.22±0.01	8.51±0.02	8.08±0.01	0.43±0.02	0.00*
24	7.17±0.01	7.04±0.03	0.13±0.02	8.22±0.04	8.01±0.02	0.21±0.02	0.05*
48	7.02±0.01	6.98±0.03	0.04±0.01	8.12±0.03	7.96±0.00	0.16±0.03	0.01*

Data are reported as means±SE. BCSCs were treated with 100 mM NaHCO_3_ for 0.5, 6, 24, and 48 h under standard culture conditions. ΔpHe is the subtraction between the corresponding pH of control medium and the pHe of BCSC culture. BCSCs: breast cancer stem cells.

* Statistically significant difference (*t*-test).

To identify the mechanism of BCSCs response to alkalinization, the expressions of mRNA CA9 and the extracellular CAe activity of the BCSCs were measured. CA9 is one of the CAe isozymes since its catalytic site faces toward the outer side of cells that catalyzes reversible hydration/dehydration reaction of CO_2_/H_2_CO_3_ (16). Adding NaHCO_3_ to the medium caused increased concentration of bicarbonate (HCO_3_^-^) ion that will further activate CAe to catalyze dehydration reaction of carbonate ion: HCO_3_^-^+H^+^→H_2_CO_3_→CO_2_+H_2_O. Increased CO_2_ concentration caused reduced pH of culture medium.

We found that the CA9 mRNA expression of BCSCs was gradually enhanced after 6-h alkalinization compared to that of non-alkalinized BCSCs (Figure 1B). A significant increase of CA9 mRNA expression was demonstrated in this study following 24- and 48-h NaHCO_3_ incubation, indicating that BCSCs responded to alkaline pHe through increased expression of CA9 gene as one of the cellular pH regulators. The increase of CA9 mRNA expression was then followed by the increased CAe activity after 6 h of incubation (Figure 1C). However, following the 24- and 48-h NaHCO_3_ incubation, the increase of CAe activity was reduced (P>0.05). The reduced CAe activity seemed to be proportional to the decrease of pHe, i.e., 8.01 for 24-h incubation and 7.96 for 48-h incubation.

### Effect of alkalinization on HIF1α gene expression and protein concentration of BCSCs

**Figure 2.**
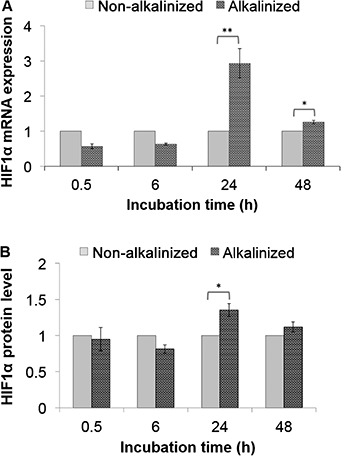
Effect of alkalinization with 100 mM NaHCO_3_ on HIF1α mRNA expression measured by qRT-PCR analysis (*A*) and protein expression of breast cancer stem cells (BCSCs) measured by ELISA (*B*). Data are reported as means±SE for n=9. Data are reported as the ratio to the non-alkalinized culture. *P<0.05; **P<0.01 non-alkalinized *vs* alkalinized BCSCs CD24^-^/CD44^+^ culture (*t*-test).

To explore the role of HIF1α in regulating the changes of pHe, we measured its mRNA and protein expression levels in BCSCs after each NaHCO_3_ incubation period. HIF1α gene expression in BCSCs was decreased up to 6-h NaHCO_3_ incubation, however, after 24-h the mRNA expression was significantly increased higher than that without alkalinization (P<0.01). After 48-h alkalinization, the increase of HIF1α mRNA was slightly decreased but still significantly higher compared to its counterpart cells without alkalinization (Figure 2A). Similar to HIF1α mRNA expression, the minor decrease of HIF1α protein level in BCSCs up to 6 h was followed by a significant increase after 24-h alkalinization. The increase of HIF1α protein level was also reduced after 48-h NaHCO_3_ incubation (Figure 2B). It seemed that alkaline pHe at a specific point might induce and activate HIF1α stabilization although under normoxia. It is known that HIF1α regulates the transcription of CA9-encoding gene under hypoxic condition (16). Nevertheless, this study demonstrated that the increase of CA9 mRNA and CAe activity was an earlier response to the alkaline pHe compared to the increase HIF1α mRNA and protein. The plausible mechanism of this phenomenon might involve the alternative signaling pathway of CA9 in response to the modulation of pHe.

### Effect of alkalinization on BCSC metabolism

**Figure 3.**
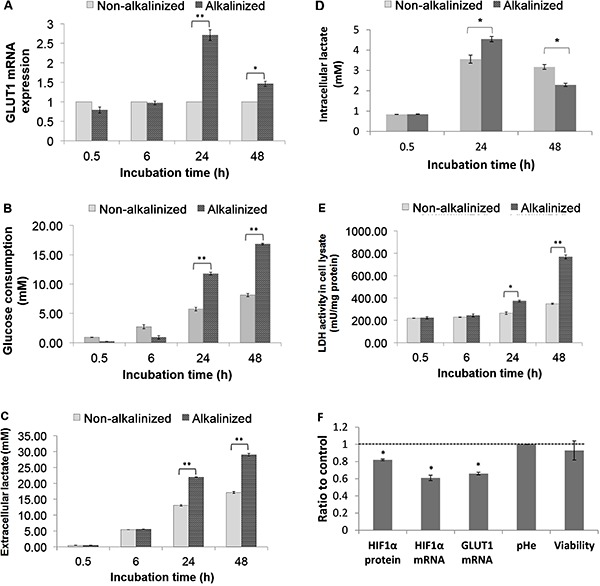
Effect of extracellular alkalinization with 100 mM NaHCO_3_ on metabolic shift towards a glycolytic phenotype in BCSCs. *A*, GLUT1 mRNA expression level analyzed using qRT-PCR. *B*, Glucose consumption of BCSCs determined by the O-toluidine colorimetric assay (at 625 nm). *C*, Extracellular lactate levels measured using a colorimetric assay and detected by spectrophotometer at 450 nm. *D*, Intracellular lactate levels measured using a colorimetric assay and detected by spectrophotometer at 450 nm. *E*, LDH activity determined using a colorimetric method and detected at 450 nm. *F*, HIF1α protein, HIF1α mRNA and GLUT1 mRNA levels, as well as pHe and viability of alkalinized BCSCs treated with FM19G11, an inhibitor of HIF1α expression. The data were normalized to the control alkalinized BCSCs with DMSO (vehicle). *P<0.05; **P<0.01, between non-alkalinized and alkalinized BCSCs (*A-E*), as well as between alkalinized BCSCs without and with FM19G11 (*F*) (*t*-test). Data are reported as means±SE for n=9. BCSCs: breast cancer stem cells; LDH: lactate dehydrogenase.

To analyze the effect of extracellular alkalinization on BCSC metabolism, we analyzed metabolic parameters including glucose consumption, lactate production, oxygen consumption, and the activity of lactate dehydrogenase. The increased glucose consumption of BCSCs following 24-h extracellular alkalinization could be clearly identified by the increase of mRNA expression encoding GLUT1 glucose transporter (P<0.01; Figure 3A).

Furthermore, the measurement of glucose level in the culture supernatant showed that the glucose consumption by BCSCs after 24- and 48-h extracellular alkalinization was significantly higher than that of their counterpart cells without alkalinization (Figure 3B). To know further about the effect of alkalinization on BCSCs glucose metabolism, we determined the lactate production in BCSCs. The results revealed that after 24- and 48-h alkalinization, extracellular lactate production in BCSCs was significantly higher than that in the cells without alkalinization (Figure 3C). In order to analyze whether metabolic shift towards a glycolytic phenotype in BCSCs causes cell death, the intracellular lactate production in BCSC lysate was additionally measured. The result demonstrated that the increase of intracellular lactate level after 24-h extracellular alkalinization was significantly higher compared to that without alkalinization (P<0.05). In addition, the colorimetric measurement also showed the increase of LDH activity in the alkalinized compared to non-alkalinized BCSC cultures (Figure 3D). Taken together, these data suggest that extracellular alkalinization may lead to the metabolic need for glucose in BCSCs. The extracellular alkalinization could increase the glycolysis in BCSCs to produce lactate and ATP. The overproduction of intracellular lactate could then contribute to the rapid decrease of pHe following the alkalinization.

To demonstrate the role of HIF1α on the regulation of glycolysis in the extracellular alkalinized BCSCs, we used FM19G11, an inhibitor of HIF1α expression. The result demonstrated that FM19G11 could significantly inhibit the expression of HIF1α at the protein, and interestingly also the mRNA level (Figure 3F), as reported in our previous study (17). The mechanism of HIF1α mRNA inhibition by FM19G11 should be further investigated. The inhibition of HIF1α protein and mRNA was in line with the inhibition of GLUT1 mRNA expression. These data confirmed the role of HIF-1α transcription factor on GLUT1 mRNA synthesis in BCSCs under alkalinized culture condition. In contrast to that, the pHe and BCSC viability were not affected by HIF1α inhibition.

**Figure 4.**
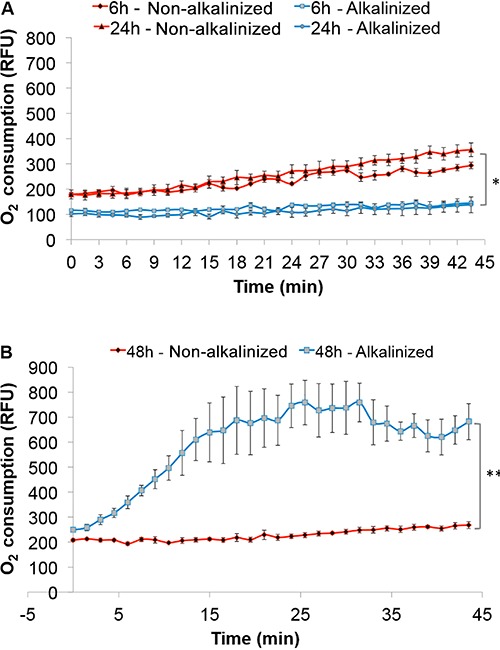
Extracellular O_2_ consumption of breast cancer stem cells after 6- and 24-h incubation (*A*) and after 48-h incubation (*B*) measured by fluorometry. RFU: relative fluorescence units. Data are reported as means±SE. *P<0.05; **P<0.01, one-way ANOVA.

The results of the extracellular O_2_ concentration of BCSCs demonstrated that O_2_ consumption of BCSCs in alkalinized medium was significantly lower than that in the non-alkalinized medium after 6- and 24-h incubation (P<0.05; Figure 4A). Contrastingly, after 48-h alkalinization the O_2_ consumption of BCSCs was drastically increased (P<0.01; Figure 4B) compared to their counterpart without alkalinization.

### Effect of alkalinization on BCSC survival

**Figure 5.**
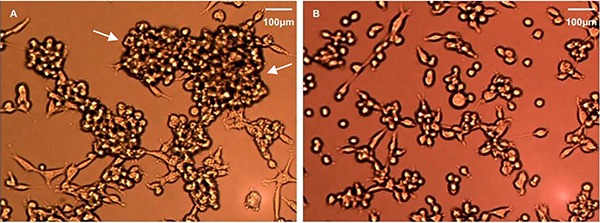
Breast cancer stem cells (BCSCs) after 24-h incubation with non-alkalinized medium (*A*) and 100 mM NaHCO_3_ alkalinized medium (*B*) observed using an inverted microscope at 10× magnification. In non-alkalinized medium, BCSCs tended to grow as cell aggregates and form mammospheres (white arrows). The number of viable BCSCs and mammospheres were reduced in alkalinized medium.

**Figure 6.**
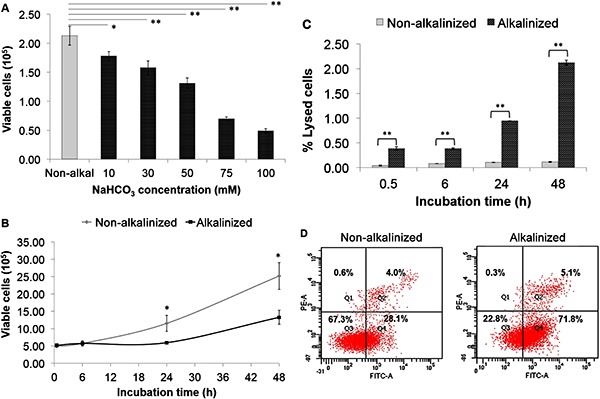
Viable breast cancer stem cells (BCSCs) measured using the trypan blue exclusion assay after 24-h incubation in cultures alkalinized with various concentrations of NaHCO_3_ (mM) (*A*). Viable BCSCs measured every 6 h until 48-h incubation in culture alkalinized with 100 mM NaHCO_3_ compared to the non-alkalinized (*B*). Percent of lysed cells determined using the measurement of % lactate dehydrogenase released into the culture medium (*C*). Apoptosis was determined using annexin V/propidium iodide assay after 24-h incubation with 100 mM NaHCO_3_. Q1: necrosis; Q2: late apoptosis and necrosis; Q3: viable cells; Q4: early apoptosis (*D*). Data are reported as means±SE. *P<0.05; **P<0.01 (*t*-test).

Under microscopic observation, we found that the morphology of BCSCs was altered in alkalinized culture. In physiologic pH, the BCSCs tend to grow as cell aggregates and form mammospheres. This kind of cell formation could not be found in cells growing in alkalinized medium (Figure 5A). Moreover, the number of viable BCSCs determined using trypan blue assay after 24-h alkalinization was gradually reduced in line with the increased NaHCO_3_ concentration in the culture medium, as shown in Figure 5B. In addition to that, we also underline that the extracellular alkalinization with NaHCO_3_ concentration ≥ 75 mM could lead to cell death since the viable cell number was obviously decreased below the inoculated cell number (Figure 6A). To confirm this result, we measured the population doubling time (PDT), LDH release, and apoptosis of BCSCs cultured in alkalinized medium with 100 mM NaHCO_3_ and incubated for 0.5-, 6-, 24-, and 48-h. Figure 6B demonstrates the growth of BCSCs in alkalinized compared to non-alkalinized medium. After 48-h culture in alkalinized medium, the growth of BCSCs was slower (13.3 x 10^5^ viable cells) compared to the non-alkalinized culture (25.2 x 10^5^ viable cells). Using the formula described in the Material and Methods section, we calculated the PDT of BCSCs to be 32.7 and 51.2 h in non-alkalinized and alkalinized culture, respectively. The PDT of BCSCs after 24- and 48-h extracellular alkalinization was significantly longer than that without alkalinization, indicating that it could also inhibit cell proliferation. In Figure 6C, we exhibit that the percentage of LDH release in alkalinized culture was significantly higher (P<0.05) compared to that without alkalinization after 0.5-h of NaHCO_3_ incubation. This suggests that extracellular alkalinization was slightly toxic to BCSCs. The apoptosis assay using Annexin V/propidium iodide resulted in the higher percentage of early and late apoptosis in the BCSCs cultured in alkalinized medium compared to that without alkalinization (Figure 6D).

## Discussion

It is known that the tumor tissue has an acidic pH that is mainly related to the metabolic activity and hypoxia (9,10). As an effort to intervene in the acidic pH of the tumor microenvironment, Robey et al. conducted a study using nude mice model, which had an implant of the cancer cell line MDA-MB-231 administered with oral NaHCO_3_ treatment. The result of their study showed that systemic NaHCO_3_ treatment increased the pHe of tumor tissue and demonstrated some therapeutic effects such as reduced number and size of cancer metastasis in the lungs, visceral organs, and lymph nodes (18). Furthermore, a study by Xie et al. (19) showed that subcutaneous NaHCO_3_ treatment to breast cancer tissue in rats, which was formed after inoculating the 4T1 breast cancer cell into the rat's mammary fat pad, caused increased intratumoral pH and necrosis.

Our study evaluated the BCSCs response to the alkaline extracellular environment in an *in vitro* study by performing alkalinization using NaHCO_3_. The response was assessed, particularly the cell capacity in regulating pH, metabolic status, and cell survival. Our study demonstrated that BCSCs gave a rapid response to the alkalinization as shown by reduced pHe from 8.84±0.00 to 8.08±0.01 after 6 h of incubation and down to 7.96±0.00 after 48 h. Although reduced pHe was also found in the non-alkalinized groups and the medium without cells, the reduction was not as fast as the alkalinized culture (Table 2).

As demonstrated in Figure 1C, the extracellular alkalinization by NaHCO_3_ supplementation in BCSC culture induced the synthesis of CA9 and the activity of CAe in order to reduce pHe. It seems that carbonate anhydrase had an active role in rapid response to reduced pHe after alkalinization as shown by increased expression of mRNA CA9, which was followed by increased CAe activity. CA9 has been demonstrated to have a role in regulating extracellular and intracellular pH as shown by a study using spheroids (20). The fast response of CA9 in regulating alkaline pHe seemed not under the regulation of HIF1α. The role of HIF1α in upregulating the expression of CA9-encoding gene appeared after 24-h incubation, when the pHe had reduced to 8.01. The results showed that the HIF1α was unlikely to play a major role in pH regulation under alkaline pHe, in particular by regulating the CA9.

Nonetheless, HIF1α seemed to play a significant role in the regulation of BCSCs metabolic shifts in alkaline conditions. This study showed that the GLUT1 gene expression pattern was similar to the HIF1α's in which the expression of both genes was increased under alkaline pHe. In this case, alkaline pHe in a specific point appeared to have a role as one of the non-hypoxic factors that may induce and activate HIF1α (21). But it should also be considered that alkaline pHe might also inhibit the HIF1α degradation, leading to HIF1α stabilization. This notion still needs to be elucidated.

The HIF1α inhibition using FM19G11 proved that the increase of HIF1α expression after 24 h extracellular alkalinization was a cellular adaptive response towards significant pHe decrease through up-regulating the expression of GLUT1, leading to the increase of glucose uptake for glycolysis. As a result of glycolysis under HIF1α regulation, the intracellular LDH activity was increased in order to catalyze the production of intracellular lactate. This process was followed by the increase of extracellular lactate level, as previously described (22). However, although the increased LDH activity after 48-h extracellular alkalinization was higher than after 24 h, the intracellular lactate level was significantly decreased in line with the decrease HIF1α and GLUT1 expression, suggesting the metabolic shift towards a more oxidative phenotype. The high increase of extracellular lactate after 48-h alkalinization indicates that intracellular lactate has been exported to extracellular compartment. Based on this analysis, we highlight that HIF1α may affect the metabolic shift towards glycolytic phenotype through the regulation of GLUT1 expression and intracellular lactate, leading to cell death (22).

The hint to metabolic inversion in BCSCs from 24- to 48-h alkalinization was also supported by our O_2_ consumption data. The HIF1α-regulated glycolytic phenotype of BCSC metabolism was related to the reduction of O_2_ consumption within 24-h extracellular alkalinization. However, this phenomenon was reversed into increased O_2_ consumption after 48-h, meaning that the BCSC metabolism was switched from more glycolytic to oxidative, as also endorsed by the data of pHe, and intracellular and extracellular lactate levels. Following 48-h incubation, pHe of BCSC culture was reduced to 7.96. It is possible that at such pHe, the BCSCs returned to their initial metabolic states, i.e. more oxidative. In addition, the high concentration of lactate in the extracellular environment could induce a backflow of lactate into the BCSCs and activate the oxidative phosphorylation. This assumption could be verified by our parallel study investigating the up-regulated expression of monocarboxylate transporter-1 (MCT1) and LDH-B genes in the 48-h alkalinized BCSCs (unpublished data, Neolaka GMG, Yustisia I, Sadikin M, Wanandi SI). MCT1 facilitates the influx of lactate into the cells, while LDH-B isozyme converts lactate into pyruvate. Another previous study has likewise denoted the occurrence of metabolic shift, in which the 4T1 breast cancer cells of rats under lactate acidosis condition became less glycolytic but more oxidative (19). It should be noted that following 48-h cell culture under physiological pHe (∼7.40), BCSCs shifted their metabolism to be more glycolytic due to a rapid cell proliferation according to the Warburg effect (23), signifying that the metabolic shift of BCSCs occurred in a distinct way under physiological conditions.

Our study showed that after 24-h alkalinization at a pH range of 7.10-8.24, BCSCs growth was inhibited starting at pHe of 7.39 following the alkalinization using 10 mM NaHCO_3_. However, at a pHe range of 7.39-7.95, there was an increased number of living cells in BCSCs, which indicated the presence of cell proliferation, although the increased number was lower than that in the non-alkalinized culture. Reduced number of living BCSCs occurred at pHe 8.07 and 8.24 following the alkalinization with 75 and 100 mM NaHCO_3_, respectively. It seemed that the death of BCSCs was initiated at pHe >8.00. The PDT of BCSCs following the alkalinization with 100 mM NaHCO_3_ was lower and needed a longer time than that of the non-alkalinized culture. Alkalinization had a low cytotoxic effect to BCSCs, only up to 2.25% of LDH was found in the medium after 48-h incubation. This result was confirmed by Annexin V/PI apoptosis assay, which demonstrated that only 0.3% necrotic cells were detected while the early and late apoptosis was 71.8 and 5.1%, respectively after 24-h incubation.

Here, we suggest that the high intracellular lactate level in BCSCs under extracellular alkalinized culture could induce acidic pHi and might be an essential cell response to reduce alkaline pHe. This response is possibly regulated by the Warburg effect through a metabolic shift towards a glycolytic phenotype. It has been reported that the acidic intracellular pH could induce apoptosis via three possible mechanisms. First, by altering the membrane potential of mitochondria; second, by activating the key enzyme that mediates the DNA fragmentation; and third, by increasing caspase-3 activation responsible for apoptosis execution (24,25). Moreover, it has been demonstrated that the mitochondria under alkaline pHe become vulnerable or unstable due to Ca^2+^ overload, leading to permeability transition, pore opening, and thus cell apoptosis (22,26).

Metabolic status shift due to alkalinization seemed to affect the survival of BCSCs. Under the conditions where the cells showed their metabolic preference to glycolytic phenotypes, cell proliferation was suppressed and cell death was induced in part. Our study revealed that alkalinization was likely to promote apoptosis rather than necrosis. Conversely, when the cells showed more oxidative phenotype as seen after 48-h culture in alkaline pHe, the cell proliferation increased. Nevertheless, we ascertained that the alteration of cell proliferation and apoptosis during extracellular alkalinization were not regulated by HIF1α. The present study implies that there may be a dynamic relationship between the changes of pHe, metabolism, and cell proliferation. BCSCs also showed a high adaptability to the changes of microenvironment pH. The role of HIF1α on the regulation of BCSCs adaptive response also needs to be considered, since it is known that under hypoxia HIF1α inhibits the proliferation of stem cells via down-regulation of the expression of c-Myc gene as a major pluripotent gene (4). Further studies are necessary to explain whether the increased HIF1α expression under alkaline pHe and normoxia may similarly down-regulate the expression of c-Myc and other stemness genes.

In conclusion, alkalinization caused increased glycolytic phenotypes of BCSCs in the form of increased glucose consumption, lactate production, LDH activity and reduced oxygen consumption, which in turn played a role in inducing apoptosis and suppressed BCSCs proliferation. Further studies are necessary to explain the correlation between alkalinization, glycolysis, apoptosis and proliferation of BCSCs. Comprehensive knowledge on this subject may become an adequate basic principle in developing target therapy, which is based on the mutual interaction between tumor microenvironment and the metabolism of cancer stem cells.
